# Defining and measuring multiple long-term conditions in research

**DOI:** 10.1136/bmjmed-2022-000287

**Published:** 2022-07-27

**Authors:** Rachel Cooper, Miles D Witham, Victoria Bartle, Avan A Sayer

**Affiliations:** 1 AGE Research Group, Newcastle University Institute for Translational and Clinical Research, Newcastle upon Tyne, UK; 2 NIHR Newcastle Biomedical Research Centre, Newcastle University and Newcastle upon Tyne NHS Foundation Trust, Newcastle upon Tyne, UK; 3 Department of Sport and Exercise Sciences, Musculoskeletal Science and Sports Medicine Research Centre, Manchester Metropolitan University, Manchester, UK; 4 ADMISSION Research Collaborative, Newcastle upon Tyne, UK

**Keywords:** Epidemiology, Geriatrics, Public health, Research design

Growing consensus in the field

In recent decades, the absolute number of people and the proportion of the population living with multiple long-term conditions (also known as multimorbidity) has increased substantially,^1^ which is a major cause for concern. Firstly, people living with multiple long-term conditions have considerably worse prognosis than those living with single conditions.^2^ This difference is especially true for people living with a combination of physical and mental health conditions,^3^ which is more often the case for younger adults living with multiple long-term conditions and those living in socioeconomically deprived areas.^4^ Secondly, people living with multiple long-term conditions typically experience a higher burden of symptoms, greater care needs, and a higher chance of decline in function and quality of life.^5^ Thirdly, many components of healthcare systems are not currently designed or equipped to deliver care efficiently and effectively for people living with multiple long-term conditions. As a result, multiple long-term conditions have been described as "one of the greatest challenges to medicine and science in the 21st century."^6^

In recognition of the scale and complexity of the challenge of multiple long-term conditions, and the urgent need to address this to improve the lives of the many people affected, there has been significant investment into multiple long-term conditions research, from underpinning biological mechanisms to pathways of care. However, progress in this burgeoning research field has been hampered by a lack of consistency in the methods used to define and measure multiple long-term conditions, which was highlighted in a systematic review by Ho and colleagues.^7^ Of 566 studies on multiple long-term conditions identified, over a third did not report a reference definition; over 10% did not list the health conditions their measure of multiple long-term conditions included; and where a list of conditions was reported, the number of conditions included ranged from two to 285. This lack of clarity and high degree of variability in definition has made it impossible to ensure comparability across studies and a solution is needed. Therefore, it is welcome news that Ho and colleagues have now, in their Delphi consensus study published in *BMJ Medicine* (doi:10.1136/bmjmed-2022-000247),^8^ developed guidance that should bring a much greater degree of consistency to the definition and reporting of multiple long-term conditions.

Ho and colleagues conducted a three round Delphi consensus exercise, involving 150 professional and 25 public participants in the first round, to ascertain how best to measure and define multiple long-term conditions in research studies. The researchers found consensus that multiple long-term conditions should be defined as the co-occurrence of two or more long term conditions, in line with definitions from the UK Academy of Medical Sciences and National Institute for Health and Care Excellence.^1 9^ Helpfully for future work, consensus (defined as ≥70% of panellists providing the same response) was also reached on five criteria that should be used to identify health conditions for inclusion in measures of multiple long-term conditions. A total of 107 conditions were presented to the panel, from which consensus was reached that 24 should always be included in definitions, with a further 35 conditions agreed to be usually included. Finally, consensus was reached on the use of simple counts versus weighted measures. Simple counts were preferred for estimating prevalence and exploring trajectories of multiple long-term conditions, and weighted measures were preferred for assessing severity of disease burden, outcome prediction, and risk adjustment. Complex multiple long-term conditions was recognised as a useful concept but no consensus was reached on how it should be defined. This is an important area for future research because of the major adverse effects on health and increased care needs related to complex multiple long-term conditions, especially at older ages.^10^

The approach taken by Ho and colleagues has considerable strengths, including involvement of the public (52% to 68% of whom were living with multiple long-term conditions in each round), and a global approach encompassing participants from multiple continents, although with a European bias. These features should ensure that the work has broad applicability and carries credibility with key stakeholders including patients, although further work to ensure that the views of a wider range of people with lived experience of multiple long-term conditions are represented would be beneficial. Some of the conditions on the "always include" list will stimulate debate, whereas other conditions surprisingly did not meet the criteria for inclusion—most notably depression, despite its high prevalence and added burden to patients and healthcare systems. Further work will be needed to ensure consistency in operational definitions for some of the 'always' include conditions (eg, paralysis), which can have considerable variation in how they are diagnosed and recorded. However, there is no doubt that the outcomes of this Delphi study represent a major step forward for the multiple long-term conditions research community and provide much needed clarity and direction.

A one-size-fits-all approach to a list of conditions is probably neither feasible nor desirable, which Ho and colleagues acknowledge. The list of conditions any group of researchers use to define multiple long-term conditions must be tailored to the research or clinical question being asked. The lists of conditions identified by Ho and colleagues do not preclude researchers from adding additional conditions as appropriate for a particular study. In fact, consensus on the five criteria for identification of long-term conditions provides research teams with a clear framework for selecting conditions in a consistent and transparent way.

We predict that Ho and colleagues’ study will soon be the reference standard for multiple long-term conditions definition and description. In our own research collaborative on multiple long-term conditions, ADMISSION, work has already begun to implement Ho and colleagues’ guidance in the context of hospitalised patients. In doing so we hope to answer their call for more multiple long-term conditions research that is comparable and reproducible. This will be essential to ensure effective translation of research into positive outcomes for people living with multiple long-term conditions.

## References

[1] Academy of Medical Sciences . Multimorbidity: a priority for global health research., 2018. Available: https://acmedsci.ac.uk/file-download/82222577 [Accessed 13 Apr 2021].

[2] Nunes BP , Flores TR , Mielke GI , et al . Multimorbidity and mortality in older adults: a systematic review and meta-analysis. Arch Gerontol Geriatr 2016;67:130–8. 10.1016/j.archger.2016.07.008 27500661

[3] Guerrero Fernández de Alba I , Gimeno-Miguel A , Poblador-Plou B , et al . Association between mental health comorbidity and health outcomes in type 2 diabetes mellitus patients. Sci Rep 2020;10:19583. 10.1038/s41598-020-76546-9 33177607 PMC7658226

[4] Barnett K , Mercer SW , Norbury M , et al . Epidemiology of multimorbidity and implications for health care, research, and medical education: a cross-sectional study. Lancet 2012;380:37–43. 10.1016/S0140-6736(12)60240-2 22579043

[5] Calderón-Larrañaga A , Vetrano DL , Ferrucci L , et al . Multimorbidity and functional impairment-bidirectional interplay, synergistic effects and common pathways. J Intern Med 2019;285:255–71. 10.1111/joim.12843 30357990 PMC6446236

[6] Whitty CJ , Oration H . Triumphs and challenges in a world shaped by medicine. Clin Med 2017;2017:537–44. 10.7861/clinmedicine.17-6-537 PMC6297683 29196355

[7] Ho IS-S , Azcoaga-Lorenzo A , Akbari A , et al . Examining variation in the measurement of multimorbidity in research: a systematic review of 566 studies. Lancet Public Health 2021;6:e587–97. 10.1016/S2468-2667(21)00107-9 34166630

[8] ISS H , Azcoaga-Lorenzo A , Akbari A . Measuring multimorbidity in research: Delphi consensus study. BMJ Med 2022. 10.1136/bmjmed-2022-000247 PMC9978673 36936594

[9] National Institute for Health and Care Excellence . Multimorbidity: clinical assessment and management. NICE guideline, 2016. Available: https://www.nice.org.uk/guidance/ng56

[10] Yarnall AJ , Sayer AA , Clegg A , et al . New horizons in multimorbidity in older adults. Age Ageing 2017;46:882–8. 10.1093/ageing/afx150 28985248 PMC5860018

